# Implementing a collaborative model in health education practice: a process evaluation of a health education programme targeting users with mental health problems

**DOI:** 10.1186/s12913-019-4819-1

**Published:** 2020-01-14

**Authors:** Regitze Anne Saurbrey Pals, Sabina Drejer, Rikke Hjort Laursen, Lone Oest, Vinie Diana Hvidbak Levisen, Naja Ramskov Krogh, Nana Folmann Hempler

**Affiliations:** 1 Steno Diabetes Center Copenhagen, Niels Steensens Vej 6, 2820 Gentofte, Denmark; 2 Consumer advocate, Skanderup, Denmark; 3 University College South Denmark, Lembckesvej 3, 6100 Haderslev, Denmark; 4 Region of Southern Denmark, Damhaven 12, 7100 Vejle, Denmark

**Keywords:** Collaborative models, Users with mental health problems, Health education, Process evaluation

## Abstract

**Background:**

Users with mental health problems (users) have a substantially higher risk of developing type 2 diabetes than the general population. Recent studies show that traditional lifestyle interventions focusing solely on exercise and diet among users have limited effect. Studies suggest collaborative models as a starting point for health behaviour change are more beneficial, but implementation in practice is a challenge. Using the Medical Research Council’s guidance for process evaluation, we explored implementation of a collaborative model in health education activities targeting users. The collaborative model focused on involving users in agenda setting and reflection about readiness to change health behaviour and was supported by dialogue tools (e.g., quotes and games). Educators received 3 days of training in applying the model.

**Methods:**

Collected data included questionnaires for users (*n* = 154) and professionals (*n* = 158), interviews with users (*n* = 14), and observations of health education activities (*n* = 37) and the professional development programme (*n* = 9). Data were analysed using descriptive statistics and systematic text condensation.

**Results:**

Ninetysix percent (152) of professionals tested the model in practice and tried at least one tool. Users reported that the model supported them in expressing their thoughts about their health and focused on their needs rather than the agenda of the professional. Ninetythree percent (143) of users strongly agreed that professionals were open-minded and responsive. However, observations showed that some professionals overlooked cues from users about motivation for health behaviour change. Furthermore, professionals identified lack of involvement from their managers as a barrier to implementation.

**Conclusions:**

Implementation of a collaborative model was feasible in practice. Training of professionals in active listening and involvement of managers prior to implementation is crucial.

## Background

People with serious mental illness have a life expectancy that is approximately 15–20 years shorter than that of the general population [1–3]. Approximately 60% of premature mortality is due to physical diseases, including cardiovascular diseases and diabetes [4]. Thus, health education is important for users with mental health problems. As a group, they are motivated to engage in health-promoting behaviour but experience barriers related to medication, symptoms of mental illness, and social isolation [5].

Lifestyle interventions targeting users with mental health problems (users) have been developed. However, the effects of traditional lifestyle interventions are limited [6, 7]. One study of a comprehensive individual lifestyle coaching intervention among people with schizophrenia and obesity found no intervention effects for any outcomes including cardiorespiratory fitness, physical activity, weight, diet, and smoking [7].

The effectiveness of traditional approaches to health education among users has been questioned [6–8]. Lifestyle interventions targeting users often focus on diet and exercise. The content and process of lifestyle interventions are primarily determined by researchers and professionals and not based on users’ preferences and needs [8–10]. A recent study showed that users did not recall being explicitly involved in physical health decisions within mental health care planning in terms of getting access to, developing, and modifying care plans [10]. This entails the risk that the needs and preferences of users related to their health and health behaviour change are neglected.

Users with mental health problems would like to be actively involved in decision-making about their health and build choices into healthy living interventions [11–13]. A meta-review of lifestyle interventions among users suggests that goal-setting, self-monitoring, and exercises that build on self-efficacy are associated with improved health behaviour outcomes [6]. Consequently, collaborative models characterised by consensus building and shared decision making have been suggested for use in this target group [12–17]. These approaches take into account the perspectives of users and professionals alike to ensure that lifestyle interventions address the needs and preferences of users [6, 8, 13].

However, implementing collaborative models in practice remains challenging [18]. Implementation is multifaceted and depends on the social and organisational context of an intervention, as well as the behaviours of those delivering and receiving it. Thus, evaluation of the processes related to intervention delivery is vital to providing insight into why the intervention might work and how it can be improved [19]. The aim of this study is to conduct a process evaluation to explore mechanisms and effects of implementing a collaborative model in health education activities targeting users with mental health problems.

## Methods

**Fig. 1:**
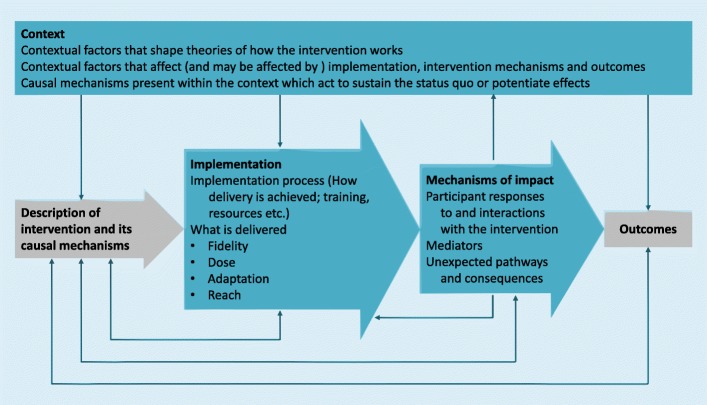
Elements of process evaluation and the relations among them according to MRC guidance. Used with permission of Moore et al. [19]

This study is part of a larger study and was guided by the Medical Research Council (MRC) framework for process evaluations of complex interventions that focus on the relations between implementation, mechanisms, and context [19] (Fig. 1). The study makes use of mixed methods as qualitative and quantitative methods were combined to increase understanding of implementation, mechanisms and context of the intervention. Qualitative and quantitative data were collected concurrently and weighted equally corresponding to a convergent study design.

### The intervention

The intervention was developed through a user-driven process actively involving users, their family members, and professionals. We applied the methodology of design thinking, which is a human-centred approach to developing interventions [20]. The design thinking process included three phases: needs assessment, ideation, and implementation. Users were involved in defining, prioritising and testing ideas for the intervention, which has been reported elsewhere [21]. This study reports findings from the implementation phase focusing on the fidelity and quality of implementation.

**Fig. 2:**
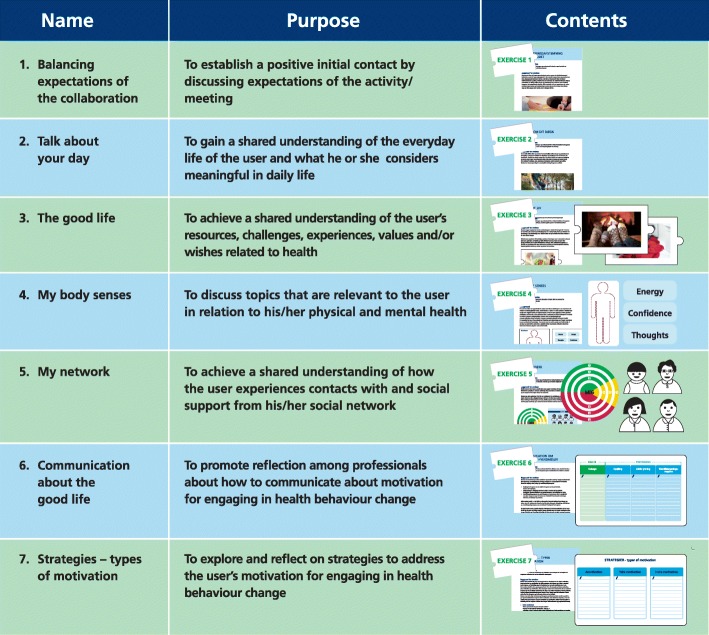
Dialogue tools to support a collaborative model

The intervention, a collaborative model, consists of an empirically grounded health education model, a guide, and seven dialogue tools to support professionals in facilitating collaboration in health education activities (Fig. 2). The health education model describes three necessary elements to facilitate collaboration about health and health-related behaviours as defined by users: 1) dialogue about the setting, 2) sharing of knowledge, 3) reflection about readiness to change. The seven dialogue tools support professionals in facilitating dialogue about the setting, sharing knowledge, and promoting collaborative reflection about readiness to change.

The collaborative model can be used in individual and group-based health education activities, such as discussions, health education, physical exercise, and cooking [22]. The collaborative model was implemented through a professional development programme conducted on 3 days over the course of several weeks. The programme target group included healthcare and social work professionals engaged in health-related activities with users in municipal and regional settings (*n* = 158). The professional development programme included a theoretical introduction to the tools, practical training with the tools, communication training through role play, and experience sharing with other professionals and users who participated in the professional development as discussion partners. Communication training included concrete techniques such as using open-ended questions, active listening, and summarising shared decisions. As part of the professional development, professionals implemented the communication techniques and at least one tool of the collaborative model in their own practices and discussed experiences with their target groups. The programme was carried out in 2016 and 2017.

**Fig. 3:**
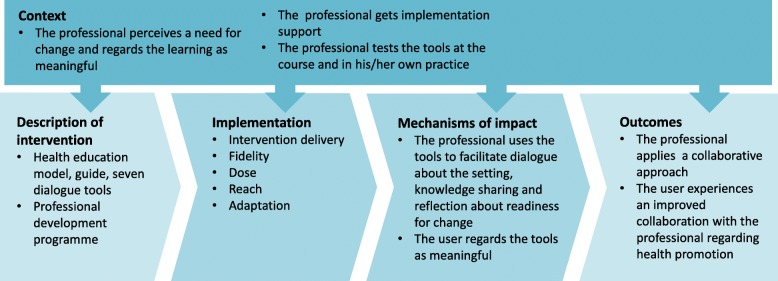
Description of the intervention based on the MRC guidance

Data collection was informed by the hypothesised mechanisms and outcomes activated by the intervention of this study, as well as contextual conditions assumed to activate the hypothesised mechanisms (Fig. 3). The hypothesised mechanisms are 1) professionals use the health education model and the dialogue tools to facilitate a collaborative approach, and 2) users regard the tools as meaningful. The described elements draw on the theoretical and empirical basis of the intervention and guided our data collection and analysis (Table 1).

**Table 1:** Types of data collected

MRC framework element	Data collection methods	Focus areas for data collection
Implementation	• Questionnaires o Users o Professionals • Qualitative interviews o Phone interviews with users • Observations o Professional development programme o Implementation in practice • Quantitative background data	• Fidelity: number of professionals who completed the professional development programme and applied the collaborative model. • Dose: number of tools used and number of times each tool was used. • Adaptation: factors associated with professionals’ use of the collaborative model, e.g., observations of professionals’ interactions with educators and users at the professional development programme and professionals’ perspectives on sharing experiences at the programme. • Reach: number of settings where the collaborative model was used and characteristics of users who participated in the intervention.
Mechanisms of impact	• Questionnaires o Users o Professionals • Observations of implementation in practice • Phone interviews with users	• Professionals’ use of the collaborative model to facilitate dialogue about the setting of the health-promoting activity, sharing of knowledge and reflection about readiness for change in users’ health behaviour. • Users’ perspectives on the collaborative model and their potential for promoting collaboration with professionals about health. This included whether users experienced being actively involved and supported in decision-making regarding their health and health-related behaviours.
Context	• Observations of professional development programme • Questionnaires o Users o Professionals	• Barriers and facilitators related to using a collaborative model in practice. In the questionnaire survey among professionals, we also examined whether they had discussed implementation with their manager and colleagues.
Outcomes	• Questionnaires o Users o Professionals • Observations of implementation in practice • Phone interviews with users	• Professionals’ use of a collaborative model. • Users’ experiences of their collaboration with professionals regarding their health. For instance, we compared data from observations and questionnaires among users which explored similar indicators of dialogue, sharing of knowledge and reflection about readiness for behaviour change.

### Observations

Between the second and third days of the professional development programme, professionals tested the collaborative model in their practices. During this approximately three-week period, we conducted observations of health education activities (*n* = 37). Observation settings (*n* = 25) were selected based on geographical distribution and type, e.g., municipal and regional. Professionals selected for inclusion were contacted by e-mail or phone with a request to participate in the study. Observations were structured around an observation guide focusing on indicators of a collaborative model.

### User interviews

We conducted interviews with users from observed settings. At the end of each observation, users were invited to participate in a telephone interview. Telephone interviews were conducted within a week after the observations so users could easily recall the use of the collaborative model and their experiences of it. We used a semi-structured interview guide focusing on users’ experiences of the collaborative model and professionals’ communication skills (Additional file 1). Interviews were audio-recorded and transcribed verbatim.

### User questionnaires

Questionnaires for users were distributed by professionals as part of testing the collaborative model between the second and third days of the professional development programme. Professionals were provided a brief guide with suggestions about how to introduce the questionnaire to users. The questionnaire, developed in collaboration with users, assessed the extent to which they agreed with experiences indicating a collaborative model, e.g., whether the professional was open-minded and responsive. Response options were agree (green smiley face), neither agree nor disagree (yellow smiley face), and disagree (red smiley face).

### Professional questionnaires

Questionnaires for professionals were collected before and after participation in the professional development programme. The initial questionnaire was distributed to professionals at the first day of the professional development programme, and the second questionnaire was collected on the last day of the programme. The initial questionnaire addressed professionals’ experience with health education and expectations for participating in the programme. The second questionnaire addressed their opinions about implementation, their overall perceptions of the professional development programme, and potential benefits gained.

### Sampling

The collaborative model was presented to professionals participating in the three-day professional development programme. Invitations to the professional development programme were given to managers in municipalities and to hospitals in Denmark, who circulated the invitation to selected employees. The participants were selected as varying in terms of organisation (municipalities and hospitals) and professional backgrounds. The professional development was carried out in three different settings to allow for geographical disparity. Interview participants were recruited through snowball sampling, where professionals and users nominated potential participants. Users were eligible if they had participated in the intervention, were > 18 years of age, and had one or more psychiatric diagnoses. Exclusion criteria were substance abuse and hospitalisation during the study period. We strived to include men and women across age groups and psychiatric diagnoses.

### Ethical considerations

The study was conducted in compliance with the Helsinki Declaration and was approved by the Danish Data Protection Agency (2012-58-0004) and processed by the Regional Committee on Health Research Ethics (H-15006076). Informed consent assuring anonymity and confidentiality was obtained from all participants. Written consent was provided for all data collection except observations and telephone interviews. Verbal consent was obtained to avoid unnecessary interruption or confusion for participants. Furthermore, written consent was not a requirement in the context of this project as the project was not characterised as a health research project according to the Danish act on ethical assessment of health research projects (LBK nr 1083 of 15/09/2017, § 2). The project adheres to the Danish executive order on information and consent in association with processing, dissemination and collection of health information (BEK nr 359 of 04/04/2019, § 2). In accordance herewith, we ensured that participant consent was voluntary, informed, specific, unambiguous and explicit. Participants’ consent was documented and audio-recorded in accordance with the guidelines of the Regional Committee on Health Research Ethics.

### Analysis

Quantitative data from questionnaires were analysed using descriptive statistics. Chi-squared tests were used to assess statistical inference. All quantitative analyses were performed in Excel. Qualitative data from interviews, open-ended responses and observations were analysed using systematic text condensation [23]. The procedure consisted of 1) reading through the material, 2) identifying and developing meaning units, 3) abstracting meaning units, and 4) reconceptualising data and developing concepts [23]. The analysis was guided by the hypothesised mechanisms, but we also looked for unexpected findings. We explored how the collaborative model was put into practice by comparing data from each health education activity (observations, interviews, questionnaire responses) with elements of the collaborative model. The results are reported according to the components of the MRC framework for process evaluations of complex interventions [19]: implementation, mechanisms of impact, context, and outcomes. Results on implementation are based on questionnaire responses, whereas insights on mechanisms of impact and context are informed by interviews, observations, and open-ended questionnaire responses. The reported outcomes are based on qualitative and quantitative data.

## Results

### Participants

Fourteen users participated in interviews. Their mean age was 37 (range, 20–57) years and six (43%) were men. Two users had no formal education, four had attended public school, five had vocational education, and two had tertiary education. A total of 153 users completed questionnaires; 87 (58%) were women, and 69 (58%, 35 missing responses) had education beyond primary school. Twenty-four (16%, 4 missing responses) of users were employed, and 108 (74%, 8 missing responses) had a psychiatric diagnosis.

One hundred and fifty-eight professionals completed the questionnaire before the first day of the programme, and 148 completed the questionnaire on the last programme day. The mean age of participating professionals was 45 years, and 132 (89%) of participants completing the program were women. Among 148 participants completing the course, 113 (76%) had prior knowledge of health education. Participating professionals had varying educational backgrounds, the most common being social work (*n* = 37), nursing (*n* = 34), and health service assistance (*n* = 27).

### Implementation

#### Fidelity

A total of 161 professionals signed up for the professional development programme and 148 (92%) completed it by taking part in at least two course days and responding to both questionnaires. Furthermore, 122 (90%, 13 missing responses) of professionals reported that the tools worked well with the target group.

#### Dose

Nearly all professionals tested the dialogue tools in their own practices, and 96% (*n* = 136) tried at least one tool. 72% (*n* = 102, 7 missing responses) of professionals reported that they significantly benefitted from the collaborative model. Neither their professional education nor their knowledge about health education influenced the extent to which they benefitted from the collaborative model (*p* = 0.35 and *p* = 0.47, respectively). Professionals with the least experience with health education were more likely to benefit from the programme (*p* = .037). Yet 59% (*n* = 20) of highly experienced professionals reported obtaining great benefit from the collaborative model.

The number of times professionals tested a single tool and the number of different tools they tested varied. Table 2 shows that professionals who tried more tools were also more likely to try each tool several times (*p* = .001).

**Table 2:** Professionals’ use of tools to support a collaborative model

Maximum tries of a single tool	Total number of tools tried		
	5–7	3–4	1–2
≥ 3	13 (65)	22 (42)	16 (25)
2	7 (35)	15 (28)	16 (25)
1	0 (0)	16 (30)	31 (49)
Total^a^	20 (100)	53 (100)	63 (100)

^a^12 participants did not respond to these items

There was no significant difference between professionals who participated in the programme in 2016 and in 2017 in the number of times they tried the tools (*p* = 0.95) or the number of different tools they tried (*p* = 0.41).

#### Adaptation

We hypothesised that professionals’ sharing of experiences with each other and with users at the professional development programme increased the possibility that they would try the dialogue tools in their practices. Accordingly, 65% (*n* = 89, 12 missing responses) of professionals reported that receiving feedback from other professionals was a valuable part of the course. We also found a positive association between professionals’ sharing of experiences and the degree to which they benefitted from using the collaborative model (*p* < .001). The positive effect of experience sharing was supported by observations of the professional development programme indicating that professionals appreciated the opportunity to share experiences with each other and users.

Professionals were very positive about user participation at the programme; 71% (*n* = 96, 12 missing responses) reported that feedback from users was a valuable part of the programme. Furthermore, we found a positive association between professionals’ perspectives on user involvement and the degree to which they benefitted from the collaborative model (*p* = .006). These findings were supported by their qualitative comments on the questionnaires, such as: “Exciting to get users’ views on things. It provided new perspectives on what works” and “The users resemble very much my target group. So I could relate their feedback to my own practice.” However, professionals reported that predefined organisational goals and agendas could hamper integrating tools into practice. Furthermore, they indicated that lack of familiarity with the tools could be a barrier to trying them. Professionals requested more time and continuous training about using the tools in practice.

#### Reach

A total of 148 professionals from 79 different settings across 21 municipalities participated in the professional development programme; 89 (60%) were from municipal psychiatric settings, 20 (14%) were from regional psychiatric settings, and 39 (26%) were from non-psychiatric settings.

Setting was not associated with the degree to which professionals benefitted from the collaborative model (*p* = 0.68) or the number of tools they tested (*p* = 0.27). Professionals reported using the tools a collective total of 655 times between the second and third course days. One hundred and fifty-three users filled out a questionnaire after trying tools with professionals; there were a total of 159 completed questionnaires because 5 (3%) users tried more than one tool and completed a second questionnaire.

### Mechanisms of impact

#### Participant responses to and interactions with the intervention

As hypothesised in Fig. 3, users reported that the dialogue tools were meaningful to them and could promote collaboration with professionals about health. Users described the dialogue tools as supporting them in articulating their resources and experiences and stimulating their ability to concentrate and stay focused during the health education activity. Users were positive about the tangible and visual elements of the tools. As one user said,

I think it was very nice to have these cards with pictures on. Instead of [the professional] saying ‘what is health is to you?’ That could be difficult to just [answer]. But having these cards made it possible to use a picture [to support the conversation].

Another user added: “When you begin to talk about issues which are a little difficult [to discuss], then you can easily lose the overview. Thus, the physical and tangible [elements] make it easier to keep the overview.”

Users described the dialogue tools as directing the focus of health education activities towards individual needs and preferences rather than the agenda of professionals. As one user noted: “I quite liked that [use of dialogue tools] instead of having a check list [a predefined agenda].” Furthermore, users mentioned that the tools could facilitate self-reflection by giving voice to their thoughts and feelings. This is indicated in the following excerpt, in which a user reflected on the use of a tool in a group activity: “Everyone [at the group activity] was not looking at me; we sat and looked at this tool. And that could be nice in terms of stepping out of it and looking at yourself in a different way ( …) it works really well for me; and I think it is a good starting point for a conversation”.

#### Mediators

As hypothesised in Fig. 3, users emphasised the significant role of professionals in facilitating collaboration and active involvement of their needs and preferences in health education activities. In interviews, users expressed that professional behaviours such as active listening, engagement and being mentally present contributed to positive experiences with using the dialogue tools. In the questionnaire survey, 93% (*n* = 140, 9 missing responses) of users strongly agreed that the professional was open-minded and responsive during the health education activity.

Users preferred that professionals provided input, asked elaborate questions and supported them in verbalising and reflecting on their thoughts. As one said: “I think it was really nice that the professionals chose some [picture] cards themselves; to show that they also were part of it. The thing that they wrote it down what we [the users] said was very nice.” Another user commented: “She [the professional] was very listening and understanding … I am not sure if one can say [that she was] provoking responses – she helped [me] to come up with answers and remember stories.” However, users indicated that professionals did not necessarily support them in translating dialogue and reflection into concrete actions to improve health-promoting behaviour in their everyday life. This was also supported by observations in which professionals mostly used the dialogue tools to facilitate dialogue and reflection about health and values among users. For instance, one user noted:

If I am to criticise something [regarding the collaborative model], it would be that the exercise only provided a snapshot [of my everyday life]. I would suggest that you [professionals] repeat the exercise every few months to see if anything has changed [in relation to users’ health behaviour]”.

#### Unexpected pathways and consequences

Fifty-six percent (*n* = 83) of professionals reported that something unexpected happened while using the collaborative model. For instance, they reported new insights about users and their perceptions about health and values. Furthermore, professionals indicated a tendency for users to become more engaged than usual in setting the agenda of health education activities and seemed surprised about their willingness to be actively involved in decisions about health. Few professionals reported negative outcomes of using the collaborative model. One professional reported that a single user felt depressed after use of the collaborative model, whereas another described a user as having difficulty concentrating.

### Context

#### Contextual factors that shape theories about how the intervention works

Observations of health education activities showed that professionals often thoroughly considered which tools they introduced and the users with whom they wanted to try them. The choice of tools seemed to depend on professionals’ preferences and confidence about using the individual tools, as well as the relationship between user and professional. Most professionals reported that they asked users about their interest in trying out the tools before observations. However, this did not necessarily include questions related to users’ preferences for the agenda, time and place of the health education activity. Few professionals encouraged users to choose tools themselves. On one hand, users did not seem to benefit from a very open-ended approach with no predetermined agenda, which resulted in confusion and indecisiveness. On the other hand, a very tight schedule left users and professionals with little room for dialogue and reflection.

Professionals reported that users’ attitudes or cognitive function could hamper the use of the collaborative model. Some professionals described the use of tools as giving rise to discussions of “difficult” issues with users, about which professionals were not confident. One professional commented, “I have been a little anxious that vulnerable users would bring something inappropriate into play [using the collaborative model].” This was supported by other professionals describing the challenge of bringing up challenging issues in relation to the Body Senses tool. However, users very much appreciated this tool, and none reported that they were uncomfortable using the collaborative model. Thus, the issue brought up by professionals might reflect their own preconceptions and worries in relation to using the model.

#### Contextual factors of implementation, mechanisms and outcomes

Only 41% (*n* = 59, 5 missing responses) of professionals reported that they discussed implementation of the collaborative model with their manager. However, 97% (*n* = 139, 5 missing responses) reported that they would like to use the collaborative model in their future work. Professionals pointed to logistical and organisational reasons for not discussing implementation with their manager, e.g., vacation and sick leave. Professionals participating in the professional development programme in 2016 were less likely to discuss implementation with their manager than those participating in 2017 (*p* = .007). This finding can be explained by the manager network meetings that were held before the professional development programme in 2017; managers were introduced to the collaborative model and organisational implications of implementation. This included the responsibility of managers for promoting professionals’ use of the collaborative model in their context.

#### Causal mechanisms present within the context

Professionals described predefined organisational targets and agendas as hampering the possibility of integrating the collaborative model into practice. One professional noted: “There are some predefined targets with the meetings [with users] which have to be met. Expectations from the system above.” Others requested time and space to integrate the approach into their practice. One professional noted that “the use of tools requires space and discretion. It can be difficult to achieve in my work.” Professionals indicated that they sometimes felt unsure about using the model and requested more training to become familiar with it. However, they also emphasised that the flexibility of the model facilitated its integration into their own practices. For instance, one professional reported that “the tools can be applied in many different ways and be adapted to the individual.” Furthermore, professionals suggested that the philosophy behind the model was compatible with their practice, as illustrated in the following comment: “It [the collaborative model] is relevant and is aligned with empowerment and the focus areas of our work.”

### Outcomes

**Fig. 4:**
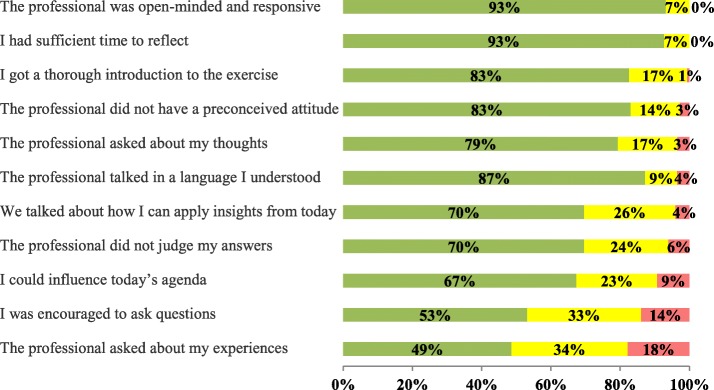
User experiences of professional behaviour

Results from the questionnaires indicated that users experienced the health education activity as a collaborative activity (mean, 2.4 on a scale from 1 “disagree” to 3 “agree”). The majority (49–93%) of users rated different aspects of professionals’ collaborative behaviour with a green smiley face, indicating that they generally had a positive experience of the model (Fig. 4). Ninety-three percent of users reported that the professional was open-minded and responsive and a similar proportion reported that they had sufficient time to reflect. However, 18% of users reported that professionals did not ask about their experiences in relation to health, and 34% reported that professionals were less likely to do so.

A majority of professionals reported that they were most likely to focus on the following elements of the collaborative model: facilitating dialogue about the setting of health education activities (64%) and sharing knowledge with users (62%). Thus, there is some discrepancy between professionals’ and users’ responses in terms of sharing knowledge about users’ health-related experiences.

Interviews with users and observations of health education activities indicated that the collaborative model was feasible in practice. Users became decisive about the content and focus of health education activities, using the dialogue tools to verbalise their thoughts and experiences. For instance, one user said, “The tools were a good way to get to know each other and make it easy to talk about your concerns.” Similarly, users reported that the tools directed focus towards their individual preferences and needs. One wrote, “It is good to ask us [users] that kind of questions. Then the staff can explore our approaches.” Another user commented, “It is exciting. I didn’t know that I could talk for so long.” However, responses to the professionals’ questionnaire indicate that they were concerned about facilitating dialogue about users’ challenges and concerns. In addition, observations revealed that some professionals tended to overlook cues from users regarding health behaviour change.

## Discussion

### Summary of findings

Using the MRC framework for process evaluation, we explored implementation of a collaborative model in health education activities targeting users with mental health problems. The majority of professionals tested the approach in their practice and tried at least one tool. Users reported that the approach supported them in expressing their thoughts regarding their health and directed focus on their needs rather than the agenda of professionals. However, some professionals were worried about facilitating dialogue about users’ challenges and concerns and overlooked their cues about motivation for health behaviour change. In addition, professionals pointed to lack of involvement from their managers as a barrier for implementation.

### Engaging professionals as change agents

Interviews and observations indicated that users appreciated the dialogue tools because they appealed to participants with a variety of learning styles and to those who otherwise might have found it challenging to become actively involved in the education process. In keeping with these findings, studies have shown that the use of pictures and game elements in health education is a promising method to facilitate person-centredness and active involvement in patient education targeting people with chronic illness [24, 25]. However, our findings also support previous reports [26] indicating that tools alone are insufficient to facilitate active involvement of the target group. Tools must be part of a collaborative relationship, not a substitute for one.

Our findings show that professionals themselves appeared to be a factor essential to facilitating collaboration in health education. Accordingly, lack of professional engagement with interventions in shared decision-making and self-management support is one of the most commonly cited barriers to this collaboration [26]. In our study, a key barrier to professional engagement with the intervention included professionals’ preconceptions about rationales for and implications of involving users in health education activities. Our findings indicate that professionals sometimes overlooked cues from users regarding health behaviour change; dialogue and reflection about health and values were almost never translated into concrete actions for health behaviour change. This suggests that professionals found it difficult to achieve an effective balance as facilitators between being directive and nondirective, which is supported by a study of professionals’ person-centred communication skills [27]. According to Cribb and Entwistle [28], this issue reflects the challenge of balancing autonomy-supportive and autonomy-undermining forms of communication.

To address the challenge of balancing autonomy-supportive and autonomy-undermining forms of communication, studies have emphasised the importance of training and supervision of professionals to generating collaboration and active participation in health education practice [17, 18, 29, 30]. This study demonstrates that professionals were able to transfer learning between the professional development programme and the context in which it was intended to be applied. According to Wahlgren [31], transfer is enhanced if professional development programmes incorporate transfer elements such as practical training, active involvement, goal setting and support and feedback from educators—all of which were elements in our professional development programme.

In addition, our professional development programme included user participation. To the best of our knowledge, no existing empirical research addresses involvement of users in professional development programmes in health education. One study shows that involvement of peer educators in facilitating education programmes for users led to increased self-confidence among peer educators and united them in helping others with mental health problems [32]. Our findings suggest that users appreciated being involved and that professionals valued feedback from and discussions with users regarding implementation. Although a few professionals remained sceptical about the intervention, the majority of professionals reported that they had gained improved collaboration and communication skills from the professional development programme. An important learning point might be that professionals retrospectively realised that they had not previously been applying a collaborative model. However, our findings suggest that the professional programme could include more training in ways to identify and respond to cues from users regarding health behaviour change. In addition, more research is needed to understand the role and value of involving users in facilitating education programmes and the optimal balance of user-professional involvement.

### Adapting the collaborative model to local context

Although good programme design is important, intervention success is also shaped by local contextual conditions. Our findings demonstrate that professionals appreciated the flexibility of the dialogue tools because it left room for interpretation and made it easier for them to individualise the content of health education activities. The flexibility of tools thus seemed to promote ownership and engagement on the part of professionals. However, professionals reported that predefined organisational targets and agendas constrained this flexibility, making it difficult for them to personalise health education activities by involving users in setting the agenda and discussing their health-related values and experiences. For instance, structured questionnaires about diet, physical activity, alcohol consumption and smoking could hamper implementation of a collaborative model. Similarly, other studies have found that professionals’ reliance on biomedical models focusing on medication and medical expertise can undermine a positive and constructive collaboration and communication between patients and professionals [33]. Furthermore, our study indicates that lack of managerial support was a barrier to implementation.

### Strengths and limitations

A key study strength is the large volume of data and the use of triangulated methods that included questionnaires, interviews and observations of health education activities and professional development programmes. Triangulation shed light on different aspects and elements of the implementation of the intervention. Triangulation also illuminated ambiguities in the data, e.g., inconsistencies between the responses of users and professionals.

A limitation of the study is that we were not able to compare data from user questionnaires before and after the intervention because they were only collected after the intervention. The Likert scale which was applied to evaluate users’ responses to the intervention might provide a limited picture as it is uni-dimensional and restricted to three options of choice. However, the scale made question answering easy and quick for the respondent, and interviews were conducted to elaborate further on users’ experiences with the intervention. Furthermore, we conducted workshops with users as part of the intervention development. Approximately three-fourths of the professionals were already using or had experience with health education methods before participating in the professional development programme. This may reflect the fact that professionals with prior experience with health education methods were more likely than those with no prior experience to participate in the programme. Finally, professionals who collected user responses were more likely than those who did not collect responses to report benefitting from the collaborative model and to test the tools in their practice.

### Implications for practice

Our findings suggest that efforts to improve health of users with mental health problems should be developed in co-creation with the target group, in terms of both development and implementation of interventions. However, implementing a collaborative model that is congruent with its underlying theoretical framework requires time and professional development. Furthermore, organisational support in terms of management commitment is crucial for sustaining the intervention.

To address sustainability, the intervention has been followed by an e-learning programme that includes an introduction to the collaborative model and case studies demonstrating the use of dialogue tools and communication techniques in practice. The e-learning programme can serve as a brush-up course for professionals and encourage experience sharing among professionals about implementation barriers and facilitators. Additional manager network meetings may help ensure that managers are introduced to the aim and organisational implications of implementing the collaborative model. Future research should include an impact evaluation exploring the effects of implementing the collaborative model using outcomes developed in collaboration with users, such as health behaviour change, quality of life and motivation for engaging in health education activities.

## Conclusions

Implementation of an empirically based collaborative model into health education activities targeting users was feasible in practice. The reach of the model was substantial, and users and professionals generally responded positively to the professional development programme and the collaborative model. A collaborative model in health education is pivotal for users’ motivation and ability to engage in health behaviour change. However, some professionals had a tendency to overlook cues from users regarding motivation for change. Furthermore, professionals pointed to organisational factors including predefined agendas and lack of involvement from their managers as barriers to implementation.

## Supplementary information

**Additional file 1.** Guide for interviews with users

## Data Availability

The data that support the findings of this study are available from Steno Diabetes Center Copenhagen, but restrictions apply to the availability of these data, which were used under the license for the current study, and so are not publicly available. However, data are available from the authors upon reasonable request and with permission of Steno Diabetes Center Copenhagen.

## Abbreviations

MRC: Medical Research Council
Users: Users with mental health problems
