# Supplementary material for: Implementing a collaborative model in health education practice: a process evaluation of a health education programme targeting users with mental health problems
Source: BMC Health Serv Res. 2020 Jan 14;20:38. doi: 10.1186/s12913-019-4819-1 (PMC6961358; doi:10.1186/s12913-019-4819-1)
Supplement: Supplementary file 1 — Additional file 1. Guide for interviews with users [file 12913_2019_4819_MOESM1_ESM.docx]

**Guide for interviews with users**

**Introduction**

In this interview, I will ask you some questions about your experiences of the health education activity that I observed with you and the professional. At the activity the professional used some new methods and tools from a development course which is developed in collaboration with users with mental health problems. We are in the process of exploring whether the tools can be used to improve the collaboration between users and professionals with regards to users’ health. Your contribution can help improve the tools. Before we start, I need to inform you that the interview is being recorded at a voice recorder and used in our research project as data material. Everything you say will be treated confidentially and only used within the context of this project. I would like to ask whether you consent to this?

**About the activity**

- What were your expectations to the activity? Did you know what you wanted to talk about with the professional?
- How did you experience the professional’s mood and engagement?
- How were you introduced to the activity?
- Did you experience that you could influence what was going to happen?
- Did you experience that you had time to think and reflect about your answers?
- How did you experience the professional’s behaviour during the activity?
  - Did the professional listen to what you said?
  - Did the professional ask about your experiences, needs and knowledge (about health)?
  - Did the professional make it meaningful and relevant to you?
- What went well? What didn’t go well?
- Was there something about the activity that you found important and can apply in your everyday life?
- Did you talk about issues that you typically wouldn’t talk about? What was that? How was that?
- Did you experience that you got new insights or that you made some decisions that could support you in the future?

**About the tools**

- Can you remember the tool/tools that was/were used?
- What did you think of the tool itself? Did the use of images and figures mean something to you?
- Follow up questions about the experience of using the tools e.g. was there something that you noticed in particular? Was there something that made a difference?

**Group activities**

- Do you know the other participants at the activity? How?
- Do you think that you had the possibility to share knowledge and experiences with each other?
- Were you encouraged to ask questions?

**Background questions**

- How old are you?
- What is your highest level of education?
- Are you working? If yes; what type of work do you do?
- Why did you participate in this activity? (mental health problem)

**Summing up**

- Was there anything about the activity that you preferred to be different?
- What is most important to you at that kind of activity?
- Overall how would you describe your experience of the activity?
